# In Vitro Confirmation of Artemisinin Resistance in *Plasmodium falciparum* from Patient Isolates, Southern Rwanda, 2019

**DOI:** 10.3201/eid2804.212269

**Published:** 2022-04

**Authors:** Welmoed van Loon, Rafael Oliveira, Clara Bergmann, Felix Habarugira, Jules Ndoli, Augustin Sendegeya, Claude Bayingana, Frank P. Mockenhaupt

**Affiliations:** Charité–Universitaetsmedizin, Berlin, Germany (W. van Loon, R. Oliveira, C. Bergmann, F.P. Mockenhaupt);; University Teaching Hospital of Butare, Butare, Rwanda (F. Habarugira, J. Ndoli, A. Sendegeya);; King Faisal Hospital, Kigali (A. Sendegeya);; University of Rwanda, Kigali, Rwanda (C. Bayingana)

**Keywords:** malaria, Plasmodium falciparum, artemisinin resistance, antimicrobial resistance, Kelch-13 gene mutations, parasites, vector-borne infections, Rwanda

## Abstract

Artemisinin resistance in *Plasmodium falciparum* is conferred by mutations in the *kelch 13* (*K13*) gene. In Rwanda, *K13* mutations have increased over the past decade, including mutations associated with delayed parasite clearance. We document artemisinin resistance in *P. falciparum* patient isolates from Rwanda carrying *K13* R561H, A675V, and C469F mutations.

Artemisinin-based combination therapies (ACTs) have contributed greatly to the global decline of illness and death from malaria (*1*). However, the novel emergence of artemisinin resistance in eastern Africa has threatened the effectiveness of these breakthrough treatments (*2*–*4*). To avert potential disaster resulting from increased resistant malaria cases, the nature and extent of this resistance in Africa urgently needs to be characterized.

Artemisinin resistance is conferred by some *Plasmodium falciparum kelch 13* (*K13*) gene mutations, only a few of which are validated markers of resistance, defined by both in vitro resistance and delayed parasite clearance in treated patients. For candidate markers, only parasite clearance applies (*1*). In Rwanda, *K13* mutations have increased over the past decade. *K13* R561H, a validated marker associated with delayed parasite clearance, was recently observed in >10% of *P. falciparum*–positive samples (*2*,*3*,*5*). In neighboring Uganda, artemisinin resistance conferred by another mutation, *K13* A675V, has recently been reported (*4*). We document in vitro artemisinin resistance in 3 *P. falciparum* patient isolates from Rwanda carrying *K13* R561H, A675V, and C469F mutations.

## The Study

We recruited malaria patients in Huye District, Rwanda, during September–December 2019 and documented patient characteristics and consent, ethical clearance, and *K13* variants elsewhere (*2*). Within 6 hours of sample collection, we cryopreserved all 66 *P. falciparum* isolates in ethylenediaminetetraacetic acid by washing the red blood cell pellet, adding freezing solution (3% sorbitol, 28% glycerol, 0.65% NaCl), and freezing at −80°C. Eight of the 66 isolates carried nonsynonymous *K13* mutations (*2*). We successfully thawed and culture-adapted 4 of the isolates in which we identified *K13* mutations: R561H, the current prevalent mutation in Rwanda; A675V, found in 11% of *P. falciparum* samples in Uganda; C469F, another candidate marker; and V555A, which is of unknown significance.

We conducted a 0–3-h postinvasion ring-stage susceptibility assay (RSA) with the active metabolite dihydroartemisinin (*6*). We exposed ring stages to a 6-h pulse of 700 nmol/L dihydroartemisinin and cultured exposed and nonexposed isolates in vitro in triplicate for 72 h. We counted parasite density per ≥10,000 red blood cells on Giemsa-stained thin blood films and calculated the means of triplicates. Dividing parasite density in dihydroartemisinin-exposed cultures by the density in nonexposed cultures provided the RSA survival rate. We considered results if 72-h growth rates exceeded 1.5× rates in the nonexposed controls and had >3 successful independent triplicate experiments per isolate. We also assessed 50% inhibitory concentrations (IC_50_) (*7*). We exposed synchronized ring-stage parasites for 72 h across a range of dihydroartemisinin concentrations (0–1 µmol/L) in duplicate or triplicate and in ≥3 independent experiments. We measured growth by SYBR Green I staining (ThermoFisher, https://www.thermofisher.com) and performed photometric assessment using FilterMax F5 microplate readers (Molecular Devices, https://www.moleculardevices.com). We estimated IC_50_ using a 4-parameter fit dose-response curve. For artemisinin-susceptible parasites, 2 cultured wild-type isolates from patients in Rwanda grew too poorly for RSA and IC_50_ assays and were replaced by artemisinin-susceptible *K13* wild-type strain NF54, which is of putative African origin. We assayed isolates in parallel with NF54 and compared IC_50_ by Student *t*-test. We performed analyses using R version 3.6.3, including the drc (dose-response curve) package (https://cran.r-project.org/web/packages/drc/drc.pdf). 

**Figure 1:**
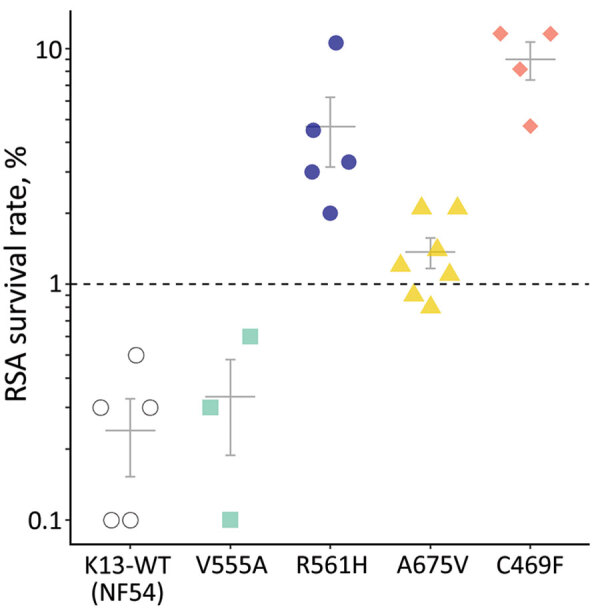
RSA 0–3-hour postinvasion survival rates (%) of an artemisinin-susceptible, *K13* WT *Plasmodium falciparum* strain (NF54) and 4 *P. falciparum* patient isolates from Rwanda with *K13* mutations. Each data point represents the mean of triplicate experiments. Isolate growth rates were only considered for analysis if 72-hour growth rates exceeded 1.5× rates in the nonexposed controls. Indicated error bars display the mean + SE; dashed line indicates the 1% survival rate threshold used to define artemisinin resistance (*1*,*6*). K13, *kelch 13*; RSA, ring-stage susceptibility assay; WT, wild-type.

**Figure 2:**
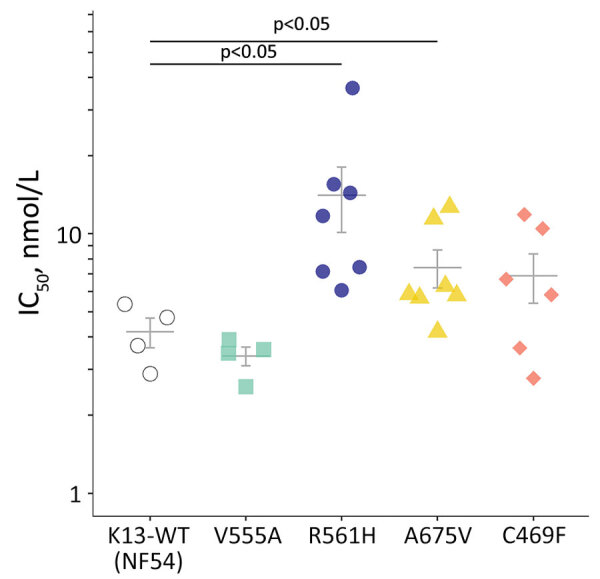
IC_50_ values for dihydroartemisinin for an artemisinin-susceptible, *K13* WT *Plasmodium falciparum* strain (NF54) and in 4 *P. falciparum* patient isolates from Rwanda with *K13* mutations. Indicated error bars display the mean + SE. p values were determined by Student *t*-test. IC_50_, 50% inhibitory concentration; K13, *kelch 13*; WT, wild-type.

RSAs yielded mean ± SE survival rates of 0.2% ± 0.1% for the NF54 strain and 0.3% ± 0.1% for V555A, well below the World Health Organization–accepted 1% resistance threshold (*1*,*6*). In contrast, 3 other isolates with *K13* mutations had >1% mean survival rates: 4.7% ± 1.5% for R561H (prevalent in Rwanda), 1.4% ± 0.2% for A675V (prevalent in Uganda), and 9.0% ± 1.6% for C469F (Figure 1). Conventional susceptibility testing yielded mean IC_50_ of 4.2 ± 0.5 nmol/L for dihydroartemisinin for the NF54 strain and 3.4 ± 0.3 nmol/L for V555A. IC_50_ levels were higher in isolates with dihydroartemisinin-resistant RSA findings: 14.1 ± 4.0 nmol/L for R561H, 7.4 ± 3.3 nmol/L for A675V, and 6.9 ± 1.5 nmol/L for C469F (Figure 2).

We determined the regional origin of the 4 tested patient isolates by single-nucleotide polymorphism (SNP) barcoding. We typed 23 SNPs to group into haplotypes associated with geographic origin (*7*,*8*). The R561H isolate displayed haplotype 9 and the other isolates haplotype 22 (*9*), confirming African ancestry.

## Conclusions

Artemisinin resistance is defined by RSA results and delayed parasite clearance in treated patients. In Africa, abundant *K13* variants circulate, but very few have been defined in terms of drug susceptibility (*1*). The *K13* mutation R561H, which has emerged in Rwanda (*2*,*5*), confers delayed parasite clearance (*3*). We found that a patient isolate with the R561H mutation from Rwanda was in vitro artemisinin resistant. Taken together, these results strongly suggest that R561H is a marker of resistance in Rwanda, a finding that needs to be confirmed in larger sample-size research. The same need for confirmation applies to *K13* candidate resistance markers A675V, recently characterized in Uganda (*4*), and C469F (*1*).

RSA survival rates for *K13* R561H *P. falciparum* in our study concord with levels in multiple gene-edited *P. falciparum* lines (*5*,*10*). Also in line with our findings are high RSA survival rates in A675V isolates from neighboring Uganda, where *K13* A675V was found in 11% and C469Y (but not C469F) in 2% of *P. falciparum* isolates collected during 2017–2019. Both mutations are associated with delayed parasite clearance (*4*). Isolates with increased survival rates also showed higher dihydroartemisinin IC_50_ levels. If this association is confirmed, IC_50_ assays that are much less labor-intensive could be useful for flagging isolates deserving additional testing by RSA.

The small number of isolates we evaluated was an obvious limitation of our study. Ideally, we would have compared the effects of individual mutations in wild-type isolates from Rwanda with study isolates, but the few selected performed poorly in vitro and were replaced by the artemisinin-sensitive NF54 strain, enabling us to verify that the RSA was working properly. A study strength is the detailed characterization of the susceptibility and ancestry of isolates.

RSA data on suspicious *K13* isolates from Africa are scarce but essential and urgent for the situational evaluation of artemisinin resistance emerging in Africa. *K13* mutations have conferred a wide range of artemisinin susceptibility when introduced in different parasite lines (*10*). Of note, artemisinin resistance identified in Rwanda and Uganda is of indigenous origin, not imported from Asia where resistance has been prevalent for years (*1*). These 2 observations argue for the need for local characterization of artemisinin resistance in circulating parasites.

Artemisinin resistance alone does not necessarily lead to ACT treatment failure, and efficacy in Rwanda still is high (*3*). However, resistance leaves the partner drug unprotected, potentially leading to resistance developing to that component as well. Eventually, this process could result in increased ACT treatment failure, which has already been observed in southeast Asia (*11*,*12*). In Africa, this development might be delayed because of prevalent partial immunity contributing to parasite elimination and high transmission increasing the likelihood of resistance allele outcrossing. Nonetheless, in Rwanda, where artemether/lumefantrine is the first-line antimalarial drug combination, a shift in the *P. falciparum* multidrug resistance 1 (*pfmdr1*) genotype pattern over the past decade suggests an increasingly lumefantrine-tolerant phenotype (*13*,*14*), although *pfmdr1* is not a validated marker for lumefantrine resistance. 

Recent research indicates that the R561H mutation is fitness neutral (*10*), implying its wider dissemination even without drug pressure. So far, a viable alternative to ACTs is not in sight. Increasing resistance, combined with the lack of effective alternative antimicrobial drugs, suggests a pessimistic scenario for sub-Saharan Africa, considering the region’s high malaria burden. Large-scale monitoring, containment strategies, and early consideration of 3-drug ACTs (*15*) are required to control widespread artemisinin resistance in Africa. 
